# Comparative Study of In Situ Gel Formulation Based on the Physico-Chemical Aspect: Systematic Review

**DOI:** 10.3390/gels9080645

**Published:** 2023-08-10

**Authors:** Insan Sunan Kurniawansyah, Taofik Rusdiana, Iyan Sopyan, Insi Farisa Desy Arya, Habibah A. Wahab, Dela Nurzanah

**Affiliations:** 1 Department of Pharmaceutics and Pharmaceutical Technology, Faculty of Pharmacy, Universitas Padjadjaran, Sumedang 45360, Indonesia; t.rusdiana@unpad.ac.id (T.R.); i.sopyan@unpad.ac.id (I.S.); 2 Study Center of Dosage Form Development Faculty of Pharmacy, Universitas Padjadjaran, Sumedang 45360, Indonesia; 3 Faculty of Medicine, Universitas Padjadjaran, Sumedang 45360, Indonesia; insi.farisa@unpad.ac.id; 4 School of Pharmaceutical Sciences, Universiti Sains Malaysia, Pulau Pinang 11800, Malaysia; habibahw@usm.my; 5 Faculty of Pharmacy, Universitas Padjadjaran, Sumedang 45360, Indonesia

**Keywords:** in situ gel, polymer, temperature triggered, pH triggered, ionic strength triggered

## Abstract

In recent years, in situ gel delivery systems have received a great deal of attention among pharmacists. The in situ gelation mechanism has several advantages over ointments, the most notable being the ability to provide regular and continuous drug delivery with no impact on visual clarity. Bioavailability, penetration, duration, and maximum medication efficacy are all improved by this mechanism. Our review systematically synthesizes and discusses comparisons between three types of in situ gelling system according to their phase change performance based on the physicochemical aspect from publications indexed in the Pubmed, ResearchGate, Scopus, Elsevier, and Google Scholar databases. An optimal temperature-sensitive in situ gelling solution must have a phase change temperature greater than ambient temperature (25 °C) to be able to be readily delivered to the eye; hence, it was fabricated at 35 °C, which is the precorneal temperature. In a pH-sensitive gelling system, a gel develops immediately when the bio-stimuli come into contact with it. An in situ gelling system with ionic strength-triggered medication can also perhaps be used in optical drug-delivery mechanisms. In studies about the release behavior of drugs from in situ gels, different models have been used such as zero-order kinetics, first-order kinetics, the Higuchi model, and the Korsmeyer-Peppas, Peppas-Sahlin and Weibull models. In conclusion, the optimum triggering approach for forming gels in situ is determined by a certain therapeutic delivery application combined with the physico-chemical qualities sought.

## 1. Introduction

The in situ gel system is a system that is liquid at room temperature but will form a gel when it comes into contact with the body or undergoes a change in pH. This form of drug delivery system is a type of mucoadhesive. Gel formation depends on several factors such as temperature modulation, pH changes, the presence of ions, ultra-violet irradiation, electrical sensitivity, and the enzyme by which the active substance is released. In situ gels are suspensions or solutions which, after reaching a specific location, undergo gelation as a result of contact with fluids within the body or physical and chemical shifts including exposure to UV radiation, external triggers, concentration of ions, pH, temperature, availability of particular molecules or ions, and others [1]. A steady plasma drug profile may be generated from an in situ gel by the use of prolonged drug release, so the medicine is absorbed effectively and attached to the gel form and can extend the drug’s lifetime within the mucosa [2].

Polymer solvents, known as in situ gels, can shift depending on the application site temperature, ionic strength or pH, and the formation of a viscoelastic semi-solid gel system. The mechanism of this formation process uses polymeric materials in response to external stimulation. Therefore, under physiological conditions, the polymer passes through a reversible phase transition, from a solution into a gel, by changing its dispersion or conformational state. By highlighting the benefits of fluids and gels, gels vary widely in terms of their application prospects pertaining to drug delivery such as oral administration, ophthalmic administration, nasal systems, and implants. In recent years, in situ gelation mechanisms have received a great deal of attention among pharmacists [3].

There are three categories of in situ gels based on their phase change performance, namely, thermally triggered systems, pH-triggered systems, and ionic-strength-triggered systems [4]. In medication delivery studies, temperature-induced hydrogel systems are one of the most widely studied types of polymer systems which are environmentally sensitive. The physiological and ambient temperatures are the most suitable range for the system’s critical temperatures, clinical handling is simplified, and gelation may be triggered without the use of any additional heat source outside the patient’s own body [5]. Gelation induced by a change in pH is another in situ gel formation method based on physiological stimulation. Every polymer that is sensitive to changes in pH has a pendant acid or base group that either accepts or releases protons in response to the surrounding pH. The term “polyelectrolytes’’ refers to polymers with an abundance of ionized groups. A slightly acidic (anionic) group in the polymer can increase external pH and cause hydrogel swelling; whereas, when the polymer contains a weak base group (cationic), external pH will decrease [6]. When cations like Mg^2+^, Ca^2+^, and Na^+^ are present, ionic activation of the gelling system occurs. In this system, changes in ionic strength cause the injected fluid to gel. This gelling system undergoes a sol-to-gel phase shift when the ionic strength is increased [7].

The mechanism of in situ gel formation can be divided into chemical and physical mechanisms. Physical mechanisms are stimulated by temperature, electric fields, and light. The chemical mechanism is stimulated by pH change and ion activation. The mechanism of in situ gel formation by pH, temperature, and ions may vary depending on the type of in situ gel system used. Here are some common mechanisms associated with these factors:

1. pH-Triggered In Situ Gel
  - pH sensitive polymers: Some polymers have pH sensitive properties, where they change from liquid to gel form when a change in pH occurs. For example, polymers such as polyacrylate, alginate, or methyl cellulose can form a gel when the pH of the solution reaches a certain threshold. Changes in pH can cause changes in the bonds between polymers, leading to gel formation.
  - Changes in environmental pH: Some in situ gels can form gels in response to changes in environmental pH. For example, in the acidic environment of the stomach, in situ drug gels containing acidic polymers will experience a decrease in pH and form a gel, which allows the release of the drugs contained in them.
2. Temperature-Triggered In Situ Gel
  - Sol-gel transition: Some polymers can undergo sol-gel transition when temperature changes occur. At a certain temperature, polymers in liquid solution form a stable gel network. For example, poly(N-isopropyl acrylamide acid) (PNIPAAm) is one of the most commonly used polymers in temperature-sensitive in situ gel systems. PNIPAAm can form a gel at temperatures above a certain “gelling temperature” called the Critical Point of Solution (LCST).
  - Gel formation by changes in ambient temperature: Some in situ gels can form when subjected to environmental temperature changes. For example, in injection applications, in situ gels may form when the polymer solution injected into the body experiences a temperature drop due to lower body temperature, thus forming a stable gel.
3. Ion Activated In Situ Gel
  - Formation of ionic bonds: Some polymers can form gels through the formation of ionic bonds with certain ions. For example, gelatin is a polymer often used in in situ gel systems, in which calcium ions are used to form stable cross-links between gelatin chains, thereby forming a strong gel.
  - Control of viscosity by ions: The addition of certain ions to a polymer solution can affect the viscosity of the solution and aid in gel formation. For example, the addition of sodium or calcium ions to an alginate solution can increase viscosity and form a stable alginate [7].

Microparticles and nanoparticles loaded with drugs may be delivered to the eye via in situ gelation mechanisms, which have several applications in ocular therapy [8]. Essential features of an in situ gel-forming technology are high viscosity and high gel strength, and the mixture should have the right viscosity to make it straightforward to administer as a solution that would quickly convert to gel [9].

One major benefit of in situ gel-forming technology over ointments is that it allows for the regular and continuous delivery of medication with a reduced risk of clouded vision. In situ gel can boost percent penetration, raise the drug’s bioavailability, and prolong sustained release in the region of application, resulting in maximal efficacy. Sustained release is a drug formulation approach that allows a medication or therapeutic agent to be delivered over time while maintaining a steady and therapeutic level of the substance in the body. It entails creating drug delivery systems that gradually release the active component over time, often resulting in increased patient compliance and decreased dose frequency. Sustained-release formulations can improve therapeutic efficacy by keeping drug concentrations within the therapeutic range for an extended length of time. This can result in improved disease control, effective management of symptoms and medical efficacy [10].

The drug contact time of sustained-release formulations is generally longer than that of immediate-release formulations. One of the main objectives of sustained-release drugs is to provide a gradual and sustained release of the drug over a longer period of time. As such, sustained-release drugs are designed to maintain therapeutic drug levels in the body over a longer period of time than immediate-release drugs. Moreover, it helps preserve the quality of medicines and makes them more stable [11].

In studies about how drugs are released, using models to understand how they are released is very important. Different models were used to explain the release behavior of API from the in situ gels in simulated gastric fluid, such as zero-order kinetics, first-order kinetics, the Higuchi model, and the Korsmeyer-Peppas, Peppas-Sahlin and Weibull models [12,13].

## 2. Materials and Methods

**Figure 1:**
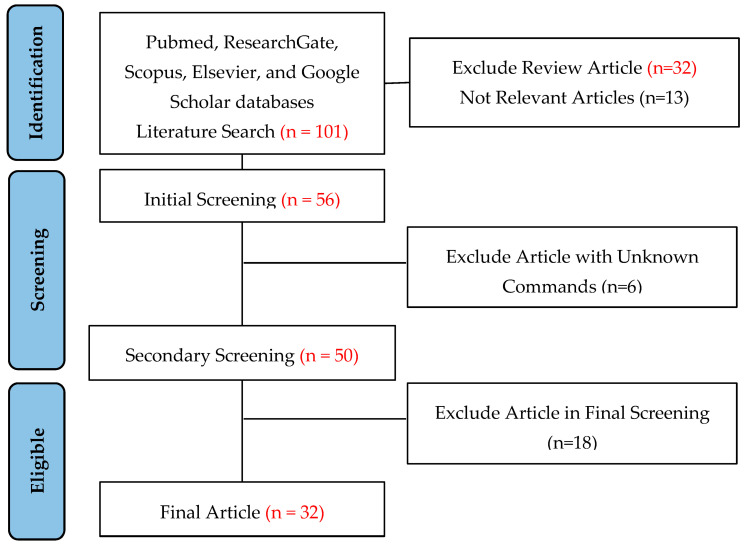
PRISMA flowchart for study selection.

We have finished the PRISMA 2020 checklist and constructed a flowchart, following the PRISMA guidelines and registration information (the registration ID is 443903). The selection process was based on the PRISMA statement 2020, and the flowchart is shown in Figure 1.

This review is based on published journals which were found using certain keywords such as ‘in situ gel’, ‘pH triggered’, ‘temperature triggered’, ‘ionic strength triggered’, ‘in situ ophthalmic gel’, and ‘in situ gel delivery system’ from various sources including the Pubmed, ResearchGate, Scopus, Elsevier, and Google Scholar databases. Opinions, reviews and irrelevant topics were excluded. Searches were quite limited to the most recent journal publications from 2020 to 2023, so there are several journals in previous years. A number of papers were used to discuss a trigger-based comparison of three types of in situ gels, and all the literature sources were included as introduction references. The best type was determined based on the research papers received.

## 3. Discussion

### 3.1. Temperature Triggered

**Figure 2:**
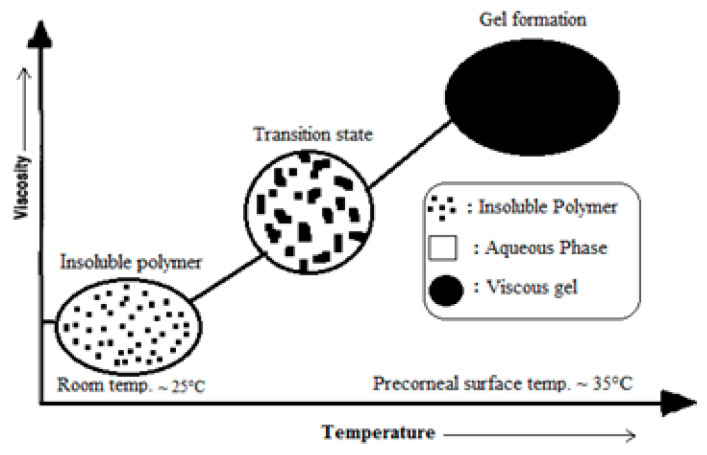
Schematic of in situ gelling mechanism activated by temperature [9] (open access).

When exposed to ambient temperature (20–25 °C), temperature-induced in situ gel-forming polymers become fluid, and at 35 to 37 °C (the physiological temperature), they begin to gel. The mechanism is shown in Figure 2 [9]. An optimal temperature-sensitive in situ gelling solution must have a phase change temperature greater than the ambient temperature (25 °C) to be able to be readily delivered to the eye; hence, the solution was fabricated at 35 °C, which is the precorneal temperature, even at concentrations lower than 5% *w*/*v*, without altering the dilution of tear fluid [14,15]. The development of hydrophobic regions and the creation of a hydrogel network from a liquid, which are both influenced by temperature, accelerate polymer chain disintegration [16]. Sol-gel transformation in the on-site gelation mechanism is caused by exposure to a temperature trigger, which is regarded as an external stimulus [17].

**Figure 3:**
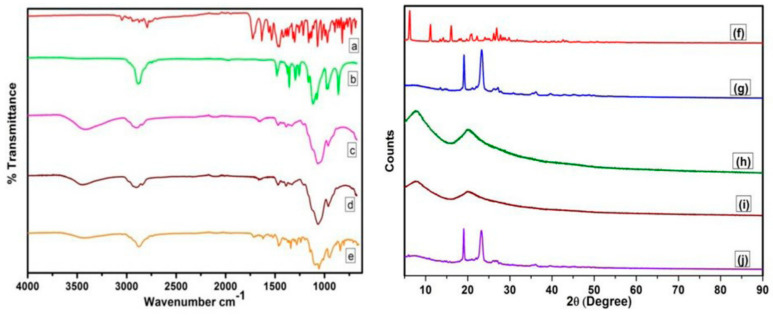
FTIR spectra of (a) ofloxacin (b) poloxamer 188 (c) HPMC K200M (d) low-viscosity HPMC (e) physical mixing of drug and polymers (h) ofloxacin PXRD spectra (f,g) PXRD spectra of the drug poloxamer 188 (h) HPMC K200M (i) PXRD spectra of low-viscous HPMC (j) PXRD spectrum of the combination of ofloxacin and poloxamer with HPMC K200M and low-viscous HPMC [18] (open access).

Balu et al. (2020) conducted an analysis of the physicochemical characteristics associated with 17 formulations that were fabricated using the cold approach, including gelling capacity, sol-gel transition, pH, flow ability, and rheological properties. Poloxamer 188, a thermoresponsive gelling agent and solubility enhancer, was used to synthesize the antibiotic ofloxacin in this study, as well as a combination of poloxamer 188 and HPMC (hydroxypropyl methylcellulose). For the purpose of extending the precorneal residence period and decreasing the frequency of dosage-form distribution, a variety of formulations were designed using various ratios. The physicochemical characteristics is shown in Figure 3. [18].

Figure 3 illustrates the comparative analysis of the physical mixture formulation of the drug and polymers, as well as the pure drug, using X-ray diffraction (XRD) peak positions. The XRD signals at 19.81° and 23.20° indicate that the physical mixture formulation exhibited a slightly more amorphous state compared to the pure drug. In the study, the researchers investigated the properties of a temperature-triggered in situ gel formulation in comparison to the pure drug. Through the analysis of XRD peak signals, it was observed that the physical combination of the formulation resulted in a somewhat amorphous structure. This finding suggests that the gel formulation may exhibit different characteristics and behavior compared to the pure drug [18].

In this study, the behavior of HPMC K200M copolymer/additives and low-viscosity HPMC blended with poloxamer 188 was investigated before and after dilution with tear fluid. The aim was to determine the critical gelling temperature of these formulations. The results showed that both HPMC K200M copolymer/additives and low-viscosity HPMC blended with poloxamer 188 exhibited higher critical gelling temperatures compared to their initial states. The critical gelling temperature for HPMC K200M copolymer/additives was found to be between 28 °C and 37.87 °C, respectively, whereas for low-viscosity HPMC blended with poloxamer 188, it was observed to be higher. These findings suggest that the addition of tear fluid influenced the gelling behavior of both formulations. The increase in critical gelling temperature indicates a stronger gel formation, which could have potential implications for their use in ophthalmic applications. Further research is needed to explore the underlying mechanisms behind this observed behavior and to evaluate the performance of these formulations in relevant biological systems. Nonetheless, these initial findings provide valuable insights into the gelling properties of HPMC K200M copolymer/additives and low-viscosity HPMC blended with poloxamer 188 in the presence of tear fluid. In the pursuit of an optimal thermos-responsive system, it is imperative to develop a formulation that exhibits fluidity at room temperature and undergoes a transition to a gel-like state upon application to the ocular surface. This desirable characteristic ensures ease of administration and enhanced contact time with the eyes, thereby maximizing therapeutic efficacy. In this proposed study, the utilization of Hydroxypropyl Methylcellulose (HPMC) as a copolymer was considered due to its enhanced biocompatibility and superior gel capacity. The primary objective was to investigate the potential of HPMC to reduce the required concentration of P188 [18].

The researchers in this study created a series of 17 unique formulations of temperature-activated in situ gels. These formulations were subsequently subjected to testing using a Brookfield Viscometer DV2T model, which was connected to a Helipath stand. The purpose of this experimental setup was to evaluate the rheological properties of the gels and assess their viscosity under varying temperature conditions. By employing this apparatus, the researchers aimed to gain insights into the gel’s behavior and determine its suitability for specific applications. Based on the research findings, it was observed that an increase in polymer concentration resulted in a corresponding increase in the viscosities of the formulations [18].

Hussain (2019) conducted a study to develop in situ gelling systems for Acyclovir utilizing temperature-triggered polymers. The formulations under investigation were observed to be in a liquid state when subjected to low temperatures. However, upon exposure to the fluid present in the tear film of the eye, a significant transformation was observed. This transformation occurred due to the presence of Pluronic F-127, a key constituent of the formulation. Specifically, the solution underwent a transition from a liquid state to a highly viscous gel state when the temperature reached 37 °C, which corresponds to the core temperature of the human body. This phenomenon highlights the potential of Pluronic F-127 to induce gelation in ocular formulations upon contact with the tear fluid, thereby offering promising prospects for ocular drug delivery systems. The impact of corneal drainage on drug bioavailability has been observed to be influenced by the viscosity of the drug in the precorneal region. Slower drainage from the cornea has been found to enhance the bioavailability of drugs. Therefore, increasing the viscosity of a drug in the precorneal region has been proposed as a potential strategy to improve its bioavailability [19].

Research by Kurniawansyah et al. (2020) aims to optimize the combined foundation of poloxamer 407 with hydroxypropyl methyl cellulose (HPMC) to discover the optimal chloramphenicol composition of in situ gels based on physical characteristics such as pH, organoleptic qualities, viscosity, and gelling ability. The optimal formula contained 10% poloxamer 407 (*w*/*v*) and 0.725% HPMC (*w*/*v*) which was designated as F6. There was no noticeable alteration in the organoleptic qualities of the F6 chloramphenicol in situ gel formulation, including its color, clarity, or scent. These findings conformed to stipulations for ophthalmic drugs. Among the results, F6 stands out as having the fastest gel formation time and the slowest melting duration. An F6 gel is created in less than 30 s and melts in more than 5 min. The pH of F6 (6.22 ± 0.02) was within the range of 5.0–7.4 which is acceptable for ocular gel formulations after 28 days of storage. Specifications for the viscosity of in situ gels in the eye were found to be 5–100 cPs, which was achieved by F6 (100.00 cPs) [20,21,22].

Polat and Unai’s studies have shown the various kinetic models that are applied mathematically to the formulations in order to examine the alendronate sodium (AS) release mechanism from the prepared combined delivery systems (zero order, first order, Higuchi, Korsmeyer-Peppas, Peppas-Sahlin, and Weibull). Six separate models were used to assess important parameters and evaluate the release kinetics of six different combination delivery systems and microsphere compositions. The kinetics of AS release from the prepared drug delivery devices were analyzed and found to fit both the Weibull and Peppas-Sahlin models, indicating that the release kinetics fit more than one model. These findings corroborate previous findings which found that the release kinetics of new drug delivery systems can match more than one model and that mixed release kinetics can be seen [13].

### 3.2. pH Triggered

**Figure 4:**
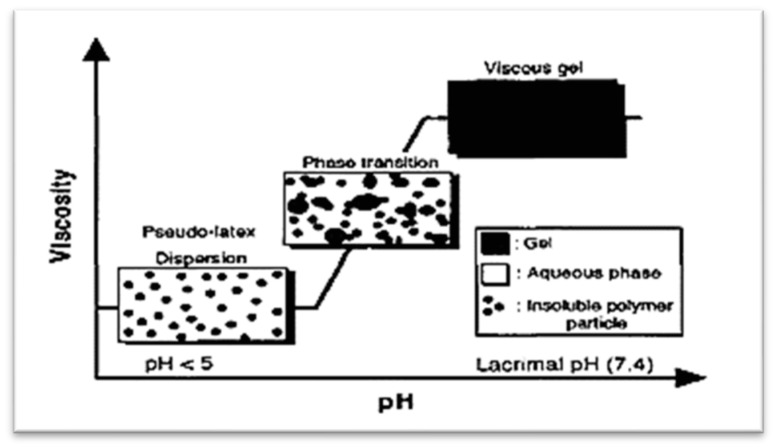
In situ gelling process triggered by pH: a schematic illustration [9] (open access).

The mildly acidic or basic groups that make up the backbone of pH-sensitive polymers respond to variations in pH by giving off or taking in protons freely. Hydrophobic, electrostatic, and hydrogen bonding interactions result in interdiffusion and conformational changes in the polymer at a specific pH, which cause the polymer to expand [23]. When the pH is increased, the polymer and mucin establish hydrogen bonds, which result in the creation of hydrogel networks. The in situ gelling process triggered by pH is shown in Figure 4 [9]. 

In a pH-sensitive gelling system, a gel develops immediately when bio-stimuli come into contact with it. Because ionized groups are present on the surface of the polymer, these systems can display abrupt changes in affinity in ions and solubility in water within specific pH ranges [15].

Gupta et al. (2008) produced several mixtures of Pluronic F-127 and chitosan and assessed their viscosity and gelling capability. The gelling and viscous properties are the two primary considerations for in situ gel formation techniques. The solution’s ideal viscosity would make it possible to instil it into the eye as drops, where the pH shift from 6.0 to 7.4 would cause the sol to quickly transform into a gel. The locally produced gel must also be stable over time, without dissolving or degrading. Chitosan (0.25% concentration) and Pluronic F-127 (9.0% concentration) were chosen because they exhibited acceptable viscosity and gelling properties. The results unambiguously showed that when the formulation’s pH was elevated and the viscosity was increased, the formulation was transformed into gel. As shown by the findings, the formula was a liquid at ambient temperature and had the desired pH level (6.0–6.2), but under the conditions of pH 7.4 tear fluid and 37 °C physiological temperature, the formulation quickly transformed into the gel phase [24].

**Figure 5:**
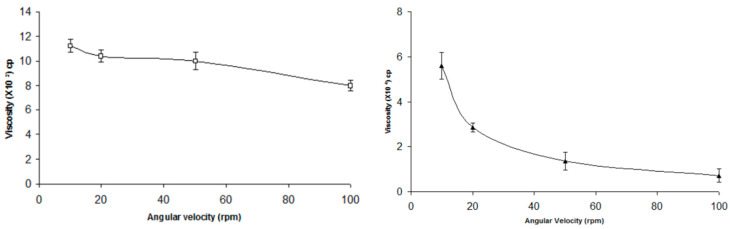
pH-activated in situ gelling system rheological profile at pH 6.0 (**left side**) and 7.4 (**right side**) [25] (open access).

In their research conducted in 2010, Gupta and Vyas focused on investigating the formulation of in situ gelation of TM. Their primary objective was to examine the gelling characteristics of carbopol-chitosan combinations at various concentrations in the STF, which had a pH of 7.4. The researchers aimed to evaluate the impact of the newly formulated gel on artificially induced elevation of intraocular pressure and also simulated tear fluid. In the course of experimentation, the enhanced formulation was subjected to shear thinning behavior, wherein an increase in shear stress resulted in a corresponding increase in angular velocity. This phenomenon can be attributed to the pseudoplastic rheology exhibited by the formulation. The liquid formulation, known as ISGF3, exhibited a low viscosity when tested at pH 6.0, as depicted in Figure 5(left side). The transformation of the liquid or solution state into a highly viscous gel was observed upon increasing the pH to 7.4, which corresponds to the pH of the standard test fluid (STF). In this study, we observed a significant finding in Figure 5(right side). The data presented in this figure provides valuable insights into the phenomenon under investigation. The specific details and implications of this finding will be further discussed.

In their study, Bharath et al. (2020) successfully developed in situ pH-triggered gel formulations incorporating BT by utilizing a combination of carbopol, a pH-activated gelling agent, and HPMC, a viscosity-enhancing compound. This innovative approach allowed for the creation of gel formulations that respond to changes in pH, providing a controlled release of BT. The utilization of in situ gelling technology in ophthalmic drug delivery has demonstrated promising results in enhancing medicine penetration within the iris and extending drug release duration for a considerable period of up to 8 h. This technology offers a unique approach to addressing the challenges associated with conventional drug delivery systems in the field of ophthalmology. By employing in situ gelling formulations, researchers have been able to achieve improved therapeutic outcomes by enhancing the bioavailability and sustained release of drugs within the targeted ocular tissues. The ability of these gels to undergo a phase transition from a solution to a gel-like state upon administration allows for enhanced drug retention and prolonged release, thereby optimizing the therapeutic efficacy of the delivered medication. The potential of in situ gelling technology to revolutionize ophthalmic drug delivery holds significant promise for the treatment of various ocular disorders, offering improved patient compliance and enhanced therapeutic outcomes. Further research and development in this field are warranted to explore the full potential. The sterility test was conducted on the mixture, and it successfully passed the test, indicating the absence of any microorganisms. Furthermore, the mixture was found to exhibit stability when stored at room temperature for a period of 60 days [26].

In the current study by Santhosh et al. (2020), in situ pH-induced levofloxacin ocular gels with varying polymer concentrations were formulated and evaluated (formulations F1 through F6). The final formulation was sterilized in an autoclave after the ocular gel was made using a straightforward dissolve procedure. When compared to the functional groups, the drug’s FTIR spectrum exhibits certain distinct peaks. There is no discernible interaction between the excipients and the medication, according to the FTIR spectrum of the physical combination of the two substances. The physicochemical characteristics and the purity of the formulation were unaffected by the final sterilization by autoclaving. All six formulations’ rheological characteristics displayed pseudoplastic behavior, with shear thinning. Prior to the inclusion of STF, from F1 through F6, the formulas’ viscosities ranged from 53 cps to 700 cps. The inclusion of STF resulted in formulations F1–F6 with viscosities between 600 and 4323 cps. According to this investigation, increasing the concentration of carbopol 934 also boosts gelling capacity [27].

### 3.3. Ion Strength Triggered

**Figure 6:**
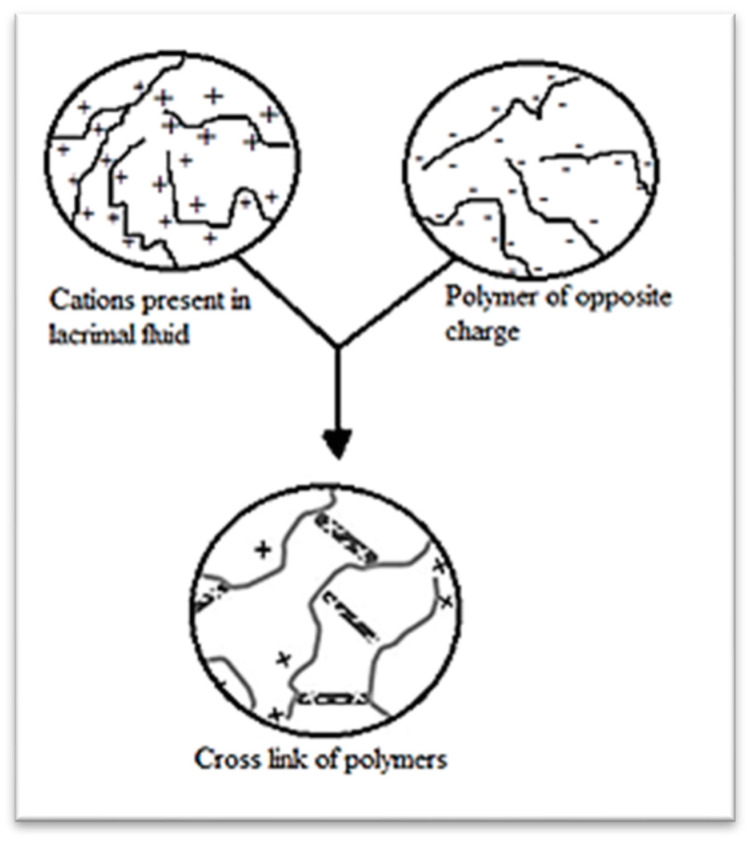
Schematic of strength in situ gelling mechanism activated by ionic [9] (open access).

In this type of gelling system, the sol-to-gel phase change happens as a result of the influence of rising ionic strength. Gels occur in lachrymal fluid due to complexation with polyvalent cations (such as Ca^2+^). The in situ gelling mechanism activated by ionic is shown in Figure 6 [9].

Among the several ion-sensitive compounds, sodium alginate sees widespread application. Deacetylase gellan gum (DGG), on the other hand, has received a lot of attention lately. Deacetylase gellan gum, a natural polysaccharide, is used by Zhu et al. in their study on an ion-activated ketotifen ocular delivery method. With increasing DGG concentration, the tested gels became viscous, but at lower concentrations (0.25% and 0.6%), a sol-gel transition of DGG caused a significant shift in viscosity. The viscosity change following gel formation was minimal with a comparatively high initial viscosity solution generated with 1.25% DGG [28].

A sustained ocular administration system for brinzolamide (BLZ) based on gellan gum was developed by Sun and Zhou (2018). For the purposes of making these formulations, varying amounts of GG (Gels A with 0.25% (*w*/*w*), B with 0.5% (*w*/*w*), and C with 1.0% (*w*/*w*)) were utilized. With increasing GG levels, the gel’s viscosity also rose. Greater viscosity changes were seen at lower concentrations (0.25 and 0.5%), and GG went through a sol-gel phase transition. On the other hand, a solution with a reasonably high initial viscosity was achieved at 1.0% GG, which meant that following gel formation, only minor variations in viscosity were noticed. The optimum batch for the next studies was the created Gel B formulation because it produced positive results. With increasing GG concentration, the test gels were more viscous. According to some theories, a denser 3D network structure is produced when the GG concentration is increased because it causes the polymer chains to become closer to one another and have more interactions with one another. When the GG concentration reached 1.0%, it became too thick and challenging to deliver using a regular nebulizer. Drug retention and bioavailability were improved by gelation because it reduced the polymers’ propensity to diffuse and degrade as well as that of the pharmaceuticals that were linked with it. According to these findings, an in situ gel-forming system preserved medication used in optical drug delivery mechanisms [29].

In a 2017 study by Nayak and Srinivasa, the gelling agent Gelrite (concentration from about 0.1 to 0.25% *w*/*v*) combined with carboxymethyl tamarind was used to create an ion-activated in situ ocular gel that included a mixture of moxifloxacin hydrochloride and ketorolac-tromethamine. A 0.2% *w*/*v* seed powder was used in the preparation of a 0.25% (*w*/*v*) enhancer. A viscosity enhancer and rate-controlling polymer at 0.45% (*w*/*v*) and an appropriately dosed preservative of benzalkonium chloride were used. The formulations were sterilized in an autoclave for 20 min at 121 °C and 15 PSI. Each and every preparation was transparent and bright yellow in color. The pH was in a range between 7.3 and 7.4. A drop of polymeric solution was placed in vials to test the gelling strength of formulations containing various ratios of Gelrite and carboxy methyl tamarind kernel powder. Prior to gelation under non-physiological conditions, all formulations were within 50 cps, and after gelation at physiological conditions, they were all within 30,000 cps. The formulations demonstrated pseudoplastic flow [30].

In their study, Calfors et al. (1998) conducted a comprehensive investigation into the rheological properties of Gelrite^®^. Gelrite^®^ is a deacetylated form of gellan gum that has the unique ability to form a gel when introduced into the eye [31]. This gelation process is facilitated by the presence of cations. The findings of this study shed light on the potential applications of Gelrite^®^ in ophthalmic formulations. In this study, the rheological properties of solutions and gels were investigated using a Bohlin VOR rheometer, manufactured by Bohlin in Lund, Sweden, which is known for its accuracy and reliability in characterizing the flow behavior of various materials. The ratio chosen for this experiment was 25 parts polymer solution to 7 parts tear fluid, aiming to mimic a scenario where a 25 mL polymer solution is mixed with the entirety of the available tear fluid, which amounts to 7 mL. The author highlights the significance of this factor in understanding and characterizing the rheological properties of different materials. The researchers investigated the effects of glycerol content on the contact times of gels with human skin. The study found that gels with lower glycerol contents exhibited longer contact times, regardless of their concentration. This finding suggests that glycerol content plays a crucial role in determining the duration of contact between gels and human skin. The study investigates the correlation between the rheological properties of gels produced in simulated tear fluid and their electrolyte content.

The viscosities of Gelrite^®^ solutions were investigated at different concentrations. To conduct the experiment, Gelrite^®^ solutions were prepared at concentrations of 0.4%, 0.6%, 0.8%, and 1.0%. The viscosity of each solution was then measured using a rheometer, which allowed for the determination of viscosity at different shear rates. By measuring viscosity at varying shear rates, the study aimed to provide insights into the flow behavior of Gelrite^®^ solutions during administration by dropping. This research contributes to the understanding of Gelrite^®^ solutions and their potential application in the field of administration by dropping. The viscosity data obtained from this study can serve as a valuable reference for future studies and the development of pharmaceutical formulations utilizing Gelrite^®^. Without the addition of salt, a gel could only be created at a 1.2% Gelrite^®^ concentration. Therefore, the 1% Gelrite^®^ solutions were easily administered as pseudoplastic liquids [32].

## 4. Conclusions

In situ gelation mechanisms are activated by temperature when the ambient temperature reaches or surpasses the LCST and employ temperature-sensitive polymers. These devices can provide prolonged drug release and have a wide range of potential uses in the medication delivery industry. Polymers sensitive to pH are used in the pH-induced in situ gelation mechanism in response to changes in pH in their immediate surroundings. These delivery methods, which allow for continuous drug release, can be employed for ocular medication. In situ gelling polymers that are induced by ion concentration may form a cross-link with cations present in tear fluid to increase drug persistence in the eye. These systems allow for numerous routes of administration and can deliver prolonged medication release. Overall, the optimum triggering approach for forming gels in situ is determined by a certain therapeutic delivery application combined with the physicochemical qualities sought.

## Data Availability

Not applicable.
